# Assessing Fluorosis Incidence in Areas with Low Fluoride Content in the Drinking Water, Fluorotic Enamel Architecture, and Composition Alterations

**DOI:** 10.3390/ijerph19127153

**Published:** 2022-06-10

**Authors:** Izabela Strużycka, Aneta Olszewska, Agnieszka Bogusławska-Kapała, Szymon Hryhorowicz, Marta Kaczmarek-Ryś, Beniamin Oskar Grabarek, Rafał Staszkiewicz, Izabela Kuciel-Polczak, Agata Czajka-Jakubowska

**Affiliations:** 1 Department of Comprehensive Dentistry, Medical University of Warsaw, 02-097 Warsaw, Poland; istruzycka@wum.edu.pl (I.S.); aboguslawska@wum.edu.pl (A.B.-K.); 2 Department of Orthodontics and Temporomandibular Disorders, Poznan University of Medical Sciences, 60-567 Poznań, Poland; anetaol@ump.edu.pl; 3 Institute of Human Genetics, Polish Academy of Sciences, 60-479 Poznań, Poland; szymon.hryhorowicz@igcz.poznan.pl; 4 Department of Histology, Cytophysiology and Embryology, Faculty of Medicine in Zabrze, Academy of Silesia, The University of Technology in Katowice, 41-800 Zabrze, Poland; bgrabarek7@gmail.com (B.O.G.); rafalstaszkiewicz830@gmail.com (R.S.); 5 GynCentrum, Laboratory of Molecular Biology and Virology, 40-851 Katowice, Poland; 6 5th Military Clinical Hospital with the SP ZOZ Polyclinic in Krakow, 30-901 Krakow, Poland; 7 Trauma Centre, Department of Ophthalmology, St. Barbara Hospital, 41-200 Sosnowiec, Poland; kuciza@interia.pl

**Keywords:** fluoride, fluorosis, oral health, fluorosis epidemiology, Dean’s Index, dental fluorosis, developmental enamel defects

## Abstract

There is currently no consensus among researchers on the optimal level of fluoride for human growth and health. As drinking water is not the sole source of fluoride for humans, and fluoride can be found in many food sources, this work aimed to determine the incidence and severity of dental fluorosis in Poland, in areas where a low fluoride content characterizes the drinking water, and to assess the impact of fluoride on the enamel composition and microstructure. The dental examination involved 696 patients (aged 15–25 years) who had since birth lived in areas where the fluoride concentration in drinking water did not exceed 0.25 mg/L. The severity of the condition was evaluated using the Dean’s Index. Both healthy teeth and teeth with varying degrees of fluorosis underwent laboratory tests designed to assess the total protein and fluoride content of the enamel. Protein amount was assessed spectrophotometrically while the level of fluoride ions was measured by DX-120 ion chromatography. The clinical study revealed 89 cases (12.8%) of dental fluorosis of varying severity. The enamel of teeth with mild and moderate fluorosis contained a significantly higher protein (*p*-value < 0.001 and 0.002, respectively) and fluoride level (*p* < 0.001) than those with no clinical signs of fluorosis. SEM images showed irregularities in the structure of the fluorotic enamel. An excessive fluoride level during amelogenesis leads to adverse changes in the chemical composition of tooth enamel and its structure. Moreover, dental fluorosis present in areas where drinking water is low in fluorides indicates a need to monitor the supply of fluoride from other possible sources, regardless of its content in the water.

## 1. Introduction

Although it is usually stated that small amounts of fluoride are essential to ensure the physiological mineralization of bones and teeth, this view is not universally held and the Scientific Committee on Health and Environmental Risks (SCHER) of the European Commission noted that, “Fluoride is not an essential element for human growth and development, and most organisms in the environment.” [1]. The research findings confirm the effectiveness and beneficial effects of low concentrations of fluoride used in the prevention and treatment of dental caries, but, on the other hand, the destructive and undesirable consequences of prolonged exposure to elevated fluoride levels [2,3,4]. For example, Cossellu et al., assessed whether local enamel fluoridation during orthodontic treatment of malocclusion might have any negative impact on the bond strength of the brackets. This type of analysis is important as patients with fixed orthodontic appliances are particularly exposed to the demineralization of tooth enamel. Based on the conducted analysis, these authors indicate that it is necessary to wait more than 15 days from fluoridation until the placement of an orthodontic appliance. This confirms the importance of assessing fluoride levels also in the context of orthodontic treatment [5]. The narrow therapeutic index for this element means that its deficiency or excess can lead to changes in teeth, bones, and skeletal structures [6,7]. However, there is currently no consensus among researchers on the optimal level of fluoride for human growth and health. The daily demand for this element, as suggested by the World Health Organization (WHO), is  <1 ppm (1 mg/L) [8], and exceeding this intake results in fluorosis, which, as reported, affects millions of people worldwide, particularly in areas where the geological composition manifests itself in a higher content of fluoride in the drinking water [9].

Dental fluorosis is a qualitative defect of enamel that occurs due to the overexposure of dental tissue to fluoride during odontogenesis. The severity of fluorosis depends on the amount of ingested fluoride, the duration of exposure, individual susceptibility, and environmental factors. Clinical dental fluorosis, depending on the severity of the condition, occurs in the form of discrete or confluent chalk-white or brown spots or lines, pits, or loss of surface enamel. The changes observed on the surface of teeth are due to the hypomineralization of the subsurface layers of dental enamel, which manifest themselves as porosity [10,11,12,13].

Nonetheless, in the broad sense of the term, dental fluorosis is no longer seen merely as an aesthetic defect. It is increasingly being emphasized that fluorosis is not an isolated symptom but instead accompanied by systemic disorders. While the effect of a fluoride supply above the norm is visible on the teeth and may appear to be a cosmetic problem only, it can lead to long latent but ultimately severe health conditions. Children with clinically diagnosed dental fluorosis also suffer from such conditions as delayed somatic development, decreased metabolic activity in bone tissue, and magnesium deficiency [14,15]. Adults with dental fluorosis are more frequently diagnosed with osteoporosis of the long bones, degenerative changes in the spine, kidney stones, thyroid disorders, and hematological changes [16,17,18].

As an epidemiological issue, dental fluorosis was first described in the early 1930s by H. Trendley Dean [19], who noted white spots on the teeth of children living in some regions of the United States. The researcher observed moderate and severe forms of this disorder in children living in areas where the water contained fluoride ions with a concentration of 2 mg/L. He also observed that a mild form of mottled enamel occurred in 2.5–43% of children when the fluoride concentration in drinking water was deficient, i.e., at a level of 0.5 mg/L.

The mechanism by which fluoride causes the progression of fluorosis is not entirely understood. It appears to be associated with delayed stripping of proteins from enamel crystals during their development phase. Unfortunately, it is still unknown whether this delay is due to an interaction with the enamel matrix proteins and proteinases, or has a direct effect on the ameloblasts. Nonetheless, eliminating the proteins is most critical during the early maturation stage of the enamel, but the highest risk of fluorosis occurs during excessive fluoride exposure in both the maturation and secretory stage [20,21,22].

The aim of this study was to assess the incidence of enamel malformations that are characteristic of fluorosis in people living in areas with low fluoride content in drinking water, as well as to assess the concentration of fluoride and total protein in tooth enamel with varying degrees of fluorosis.

## 2. Materials and Methods

### 2.1. Population Study

The study population comprised 696 generally healthy individuals aged 15 to 25 years seeking dental care. The patients had lived from birth in areas (Greater Poland region) where the fluoride concentration in drinking water was low, i.e., not exceeding 0.25 mg/L. The presence or absence of dental fluorosis was recorded during the oral examinations for each patient, based on widely adopted evaluation criteria; namely, this includes generalized (affecting more than two teeth), symmetrical lesions in homonymous teeth in the form of spots of varying shape and size, either of a white or, in more advanced cases, brown shade. Abnormal mineralization in the form of single, oval, non-translucent, or asymmetrical lesions constituted an exclusion criterion for the study group. The severity of the condition was evaluated using the traditional criteria (Dean Index, http://www.fluorosisindex.com/deansindex, accessed on 12 March 2022) [19], and thus the study included a fluorosis assessment of the two teeth with the most severe lesions.

### 2.2. Qualification for the Study

The criterion of inclusion in the clinical study was the state of the dental crowns of patients. The patients who qualified for clinical evaluation were those with healthy enamel and with hypomineralization changes of various degrees. Fluorosis is defined as developmental changes in the enamel, which develop during the formation of hard structures of the tooth during its development, and when using oral cavity hygiene measures with fluoride does not worsen the clinical state. Clinical studies were conducted in areas with a low content of fluoride in the drinking water, while teeth for the in vitro study were obtained from areas with fluoride ion content. Fluorosis was more prevalent in these areas and thus the material for studies was easily available. On the other hand, in order to diagnose the severity of fluorosis, a diverse level of fluoride in the water is of no consequence, since most likely the incidence of fluorosis in areas with low content in the drinking water is a result of the combination of the influence of other fluoride sources, such as air, nutrition, and hygiene measures.

### 2.3. In Vitro Analysis of Extracted Human Teeth

The study included 30 healthy premolars without active caries that were extracted for orthodontic reasons. Group I comprised 10 teeth without any changes, typical of abnormal enamel development. Group II consisted of 10 teeth with lesions typical of mild fluorosis, obtained from patients living in areas with a low fluoride concentration in drinking water. Group III included 10 teeth with moderate fluorosis, obtained from patients living in areas where fluoride concentrations in drinking water ranged from 0.8 to 1.2 mg/L. Before analyzing the protein and fluoride content in enamel, the teeth were washed in an ultrasonic cleaner, and those intended for protein analysis were additionally rinsed with 70% ethanol to remove any proteins present on the enamel surface [20]. Next, to facilitate the dissection of the enamel, a longitudinal cut was made across the crown of each tooth, running from the buccal surface towards the lingual surface. The cuts were made using a low-speed saw with a 0.1 mm diamond disc. Surface images of randomly selected sections were obtained with a scanning electron microscope (SEM).

#### 2.3.1. A Quantitative Analysis of Total Protein Content in Tooth Enamel

The dissected enamel from each tooth was ground down, weighed, and mineralized in a mixture of 4 M guanidine-HCl and 0.5 M EDTA for 7 days. Next, DC Protein Assay (Bio-Rad, Hercules, CA, USA) was used. To construct the standard curve, serial dilutions of bovine serum albumin were prepared in concentrations between 14 and 0.43 mg/L in test tubes. An amount of 500 mL of reagent A, followed by 4 mL of reagent B, was added to each tube containing 100 µL of serial dilutions of the reference standard solution, After the solutions were mixed in a vortex mixer and incubated at room temperature for 15 min, absorbance was measured at a wavelength of 750 nm using a UV-VIS DU 640 spectrophotometer (Beckman Coulter, Brea, CA, USA). In order to determine the amount of protein in the assessed samples, test tubes were filled with 100 µL of solutions obtained through the mineralization of human tooth enamel from the study groups. An amount of 500 µL of reagent A, followed by 4 mL of reagent B, was added to each tube Then, the protein concentration was measured. The total protein content was determined from the standard curve and converted into milligram units of enamel protein per gram of enamel.

#### 2.3.2. Determination of Fluoride Content in Tooth Enamel

Enamel dissected from each tooth was ground into a powder in an agate mortar, then weighed and mineralized, using HNO_3_ and H_2_O_2_ (Merck, Darmstadt, Germany) at 80 °C for 3 h. All the solutions were prepared in deionized water with a resistivity of <18 m MΩ.cm (at 25 °C) using the Milli-Q system (Millipore, Molsheim, France). The standard fluoride (F^−^) solution was prepared by diluting a solution of 1000 mg F^−^/L (Merck). The mobile phases were prepared from appropriate aliquots of the following salts: Na_2_CO_3_, and NaHCO_3_ (POCh, Gliwice, Poland). The F^−^ anions were separated by DX-120 ion chromatography (Dionex, Sunnyvale, CA, USA) with conductivity detection. The eluent was a mixture of Na_2_CO_3_/NaHCO_3_, administered in isocratic mode at a 1 mL/min flow rate. The fluoride content was determined from the standard curve and converted into milligram units per enamel gram.

#### 2.3.3. Scanning Electron Microscopy (SEM)

Randomly selected sections of the teeth were treated with 30% phosphoric acid for 30 s before the microscopy procedure [23]. The prepared enamel specimens from groups I and III were placed on specially prepared blocks coated with a resin layer to prevent the movement of the samples during the examination. Each sample was coated with a layer of gold in a Denton Vacuum Desk II sputter coater. Images of the surfaces and cross-sections of the tooth enamel were taken using a scanning electron microscope (Hitachi, Japan). The images were taken at ×450 and ×1600 magnification.

### 2.4. Statistical Analysis

#### 2.4.1. Sample Size Calculation

According to the data published by the Central Statistical Office (GSO) in 2020, the number of inhabitants of the Greater Poland Voivodeship was 3,496,500 [24]. The number of participants in the study was determined using the statistical tool available at https://www.naukowiec.org/dobor.html (accessed on 12 March 2022) [25]. For a population of 3,496,500 inhabitants, the maximum error value was estimated at 7%. Therefore, assuming a *p*-value < 0.05, the required number of respondents in the study was 180. In turn, for a population of 38,350,000 inhabitants, the maximum error value was estimated at 7%, and the required number of participants in the study was 196 (*p*-value < 0.05).

#### 2.4.2. Experimental Groups Comparison

The normality of the distribution and the homogeneity of variable variances tests were conducted in the experimental groups using the Kolmogorov–Smirnov test and Levene’s test, respectively. Studied groups were compared using a one-way ANOVA, and pairwise comparisons were performed using the post hoc Tukey’s HSD (honestly significant difference) procedure. As being indicative of statistical significance, we considered *p*-values below 0.05. Statistical calculations were made using Social Science Statistics webpage [26].

## 3. Results

### 3.1. Findings of the Population Study

In the 696 examined teenagers and young adults living in areas with low fluoride content in drinking water (below 0.25 mg/L), dental fluorosis of different severity levels was present in a total of 89 cases (12.8%), comprising 51 women (57.3%) and 38 men (42.7%). Fluorosis severity level was assessed and characterized according to the Dean’s Index (http://www.fluorosisindex.com/deansindex, accessed on 12 March 2022). We observed 12 cases (13.5%) of questionable fluorosis (code 1, with slight aberrations from the translucency), 56 cases (62.9%) of very mild fluorosis (code 2, 10–25% of surface) and 21 cases (23.6%) of mild fluorosis (code 3, 25–50% of surface) (Table 1). We did not detect moderate or severe fluorosis in the study group (codes 4 and 5; Table 1).

### 3.2. In Vitro Studies of Extracted Teeth Enamel

#### 3.2.1. Quantitative Analysis of Total Protein in Tooth Enamel

When analyzing the assessed samples of tooth enamel obtained from teeth with different stages of fluorosis, we found statistically significant differences in the total protein content (*p*-value = 0.002). The descriptive statistics, including the means with standard deviations (SD) and *p*-values for pairwise comparisons, are presented in Table 2.

The analysis revealed that the enamel of teeth with mild fluorosis (group II) and with moderate fluorosis (group III) contained a significantly higher content of protein when compared to the enamel of teeth with no clinical signs of fluorosis (group I) (*p* = 0.002 and <0.001, respectively). No statistically significant differences were recorded when comparing groups II and III; however, the protein level in the moderate fluorosis group was slightly higher than in the mild fluorosis one.

#### 3.2.2. Fluoride Content in Tooth Enamel

The analysis of the fluoride content in the enamel of the examined teeth showed that the enamel of teeth with mild (group II) and moderate fluorosis (group III) contained a significantly higher amount of fluoride than teeth enamel with no developmental changes (group I) (*p*-values < 0.001). The difference between groups II and III was noticeable but statistically irrelevant (*p*-value = 0.221). The fluoride content in studied groups was presented as means with standard deviations, and *p*-values obtained for pairwise comparisons are presented in Table 3.

#### 3.2.3. Findings of the SEM Examination

**Figure 1:**
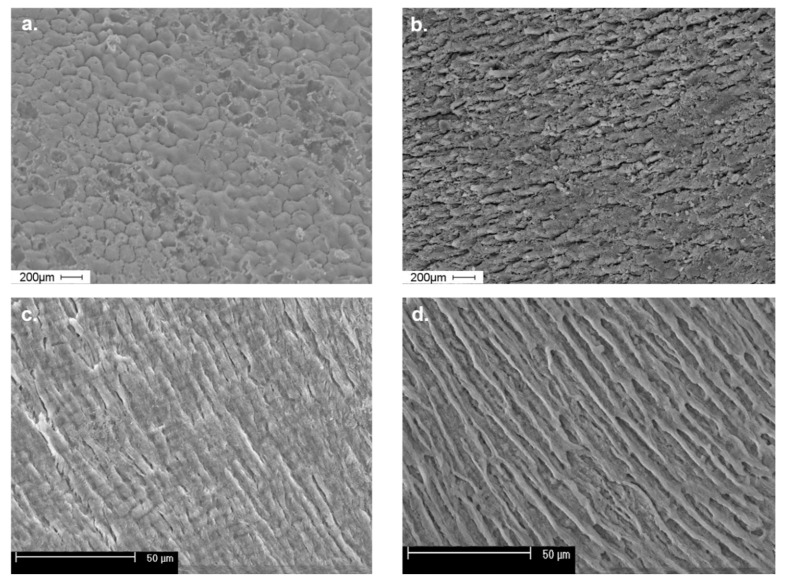
Scanning electron microscopy (SEM) imaging in a healthy and fluorotic tooth: (**a**) buccal enamel surface without fluorosis; (**b)** buccal enamel surface with fluorosis; (**c**) cross-section of enamel without fluorosis; (**d**) cross-section of enamel with fluorosis. Scale is shown in the left bottom corner of each image.

We assessed the buccal surfaces and cross-sections of tooth enamel of healthy premolars and those with moderate fluorosis signs (Figure 1).

Enamel surfaces in which no fluorotic lesions were detected (a) had a regular prismatic structure where the interprismatic substance separating single hexagonal enamel rods was characterized by marked boundaries. The enamel rods observed in the enamel cross-section were arranged in a regular, organized, and parallel pattern and featured a visibly narrow rod sheath (c).

The surface of tooth enamel with moderate fluorosis lacked a regular prismatic structure and surface smoothness (b). The image shows areas with an uneven structure as well as linear fissures, which make the enamel surface rough. Similarly, a cross-section of the enamel (d) reveals a loss of surface smoothness and regular and longitudinally arranged mineralized prism-like structures, whose transverse dimension is smaller than that of the prisms in the healthy tooth (c). Wide and deep spaces can be observed between the prisms along with irregular structures.

## 4. Discussion

We found that fluorosis is present and even more frequent than we expected in teenagers and young adults living in areas with low fluoride content in the drinking water. In the study group, 12.8% of the participants experienced an abnormal mineralization of hard dental tissue in the form of mottled enamel. We also observed a prevalence of fluorotic changes in females (57.3 vs. 42.7%). In the in vitro studies, we detected statistically relevant differences in fluoride and protein levels between healthy and fluorotic premolars enamel. It is worth emphasizing that the differences in fluoride and protein levels between mild and moderate fluorosis were noticeable but statistically not significant, which indicates that even a slight rise in fluoride affects the macroscopic picture of fluorosis. On the other hand, essential and statistically relevant differences between healthy teeth and mild fluorotic ones indicate a relatively high level of fluoride needed for the first clinical symptoms to occur. It is possible that the level of systemic fluoride may be higher than is apparent in the form of changes in teeth enamel, which is potentially threatening.

Dean’s observations from the 1930s [19] started research that was focused on fluorosis, proving a correlation between the incidence and severity of fluorosis and fluoride concentrations in drinking water [27,28,29,30,31,32]. However, further research in this field revealed that the disorder also occurs in areas where the fluoride ion content in water is low [33,34,35,36]. The current state of knowledge regarding the origin of this developmental disorder states that even low fluoride concentrations can adversely affect enamel formation if supplied during the critical period of amelogenesis [37].

The findings obtained in our study confirmed reports in the international literature on the prevalence of fluorosis in areas with a low fluoride content in the drinking water. The findings of several epidemiological studies confirm the global increase in the incidence of mottled enamel [38,39,40,41,42,43]. However, water consumption with increased an fluoride content accounts for only 40% of fluorosis cases; the remaining 60% are connected with fluoride intake from other sources [44]. The optimum level of fluoride exposure is, in fact, often exceeded due to the concurrent ingestion of fluoride ions from various sources [45,46,47,48].

The substantial increase in fluorosis observed in recent years is associated with the emergence of many additional, essential sources of fluoride, which influence the human body [39,49]. Some authors believe that the introduction of brushing with fluoride toothpaste on a widespread and daily basis was the main factor behind the increased prevalence of this disorder. The lack of adult supervision of children when brushing their teeth with fluoride toothpaste and the excessive use and even swallowing of such toothpaste continue to be risk factors for dental fluorosis, as documented in research [50,51]. The observations of Butera et al., are also interesting. They have shown that in the case of patients with composite dental restorations, a good solution is the daily use of a toothpaste containing Zn-carbonate hydroxyapatite [52].

The latest reports from North Columbia, which is a region with low fluoride content in the drinking water and fluoridates salt as part of the public health prophylactic program, concerning a three-year follow-up study in 8–12-year-old children, showed the dynamic post-eruptive nature of this dental fluorosis and a significant proportion of the teeth with more advanced fluorotic changes [53]. The fact that no cases of severe fluorosis were recorded in this study indicates that the study population had not been exposed to high concentrations of fluoride during amelogenesis. Research conducted worldwide in areas with low fluoride content is consistent with the findings of the present study. In American studies carried out as part of the National Health and Nutrition Examination Survey (NHANES) 2015–2016 concerning 2098 children and adolescents, the fluoride concentration in water above the level of 0.7 mg/L was 25%, but the prevalence of dental fluorosis was 70%. In this study, higher plasma fluoride concentrations were associated with higher odds of dental fluorosis in females, while this association almost disappeared in males [54]. In the presented research, we noticed the prevalence of fluorosis in females, which may suggest an influence of gender-specific hormonal and mineral metabolism.

In a recent literature review, Jullien summarizes that the early childhood intake of fluoride supplements and fluoride in drinking water helps to prevent dental caries but is associated with an elevated risk of dental fluorosis ranging from minor to severe forms. While this study recognizes the scale of the problem, it only considers fluorosis as a cosmetic defect [55], which in our view, underestimates its underlying molecular basis.

In vitro studies revealed that fluoride alters mitochondrial homeostasis and dynamics via alterations in reactive oxygen species (ROS), ATP, cytochrome C, and electron transport complex proteins synthesis, leading to cell apoptosis. Even a low dose of fluoride exposure induces ROS synthesis, which causes further damage to the mitochondrial membrane potential. Furthermore, a high concentration of fluoride induces apoptotic signaling, causing the expression of pro-and anti-apoptotic genes [18]. Other findings showed that fluoride treatment induces apoptosis through the mitochondrial p53 pathway, specifying the involvement of p53-dependent transcriptional activity. Moreover, fluoride deregulates the expression of the *Sirt1* gene, inducing the p53-mediated apoptosis pathway upregulation, which indicates that *Sirt1-*p53 could be a potential target in fluorosis treatment [56]. A high level of fluoride also promotes apoptosis through the endoplasmic reticulum (ER) stress pathway. A fluoride treatment in ameloblast cell lines induces the upregulation of the unfolding protein response, leading to ER stress and enamel protein accumulation [57]. Seeing as how the whole body fluoride level reflects irregularities in teeth enamel, dental fluorosis should neither be treated as being simply a cosmetic defect nor be underestimated.

In all enamel samples examined with SEM imaging, which displayed any characteristic of fluorosis irregularities in the prismatic structure, the recorded fluoride content was significantly higher than in samples with a regular structure of the enamel rods. The analysis showed a higher fluoride content in the enamel of fluorotic human teeth than in healthy teeth enamel. The findings of studies conducted by other authors suggest a link between the presence of clinical symptoms of fluorosis and increased fluoride content in enamel, which in the case of erupted teeth, accumulate predominantly in the outer third of the enamel, reaching its highest values on its surface [58,59,60,61]

The mechanism for fluorosis development is highly complex, and proteins, particularly amelogenins, may play a vital role in this process. The research findings presented in the present text revealed a difference in protein content between enamel with clinically diagnosed moderate or mild fluorosis and normal enamel. These findings are consistent with the research conducted by Wright et al. [20], which confirmed a significantly higher protein content in fluorotic enamel. Earlier reports by Aoba and Fejerskov [62] suggested qualitative rather than quantitative changes in protein content, indicating a residual organic matrix containing proteins similar to amelogenins, rich in proline, glutamic acid, and aspartic acid, and histidine.

To summarize, even mild forms of fluorosis result in changes in the chemical composition of enamel, and in a moderate form, such changes become more severe, resulting in irregularities in the structure of tooth enamel. It is alarming that the incidence of fluorosis has increased in recent years. If the supply of fluoride remains uncontrolled, fluorosis may occur in more severe forms in the future, causing widespread aesthetic defects and not yet fully recognized systemic disorders. Research is specifically needed to determine the prevalence of dental fluorosis in fluoridated and non-fluoridated communities regarding fluoride intake with food and widely available dietary supplements. Moreover, campaigns to raise public awareness of fluorosis are needed.

Overdosing fluoride compounds in adults is more probable in regions with a high level of fluoride in the drinking water than in regions with artificially fluorized water. On the other hand, the risk for children is greater at a young age due to swallowing toothpaste [63]. The incidence and strengthening of dental fluorosis depend on the parameters of the exposure to fluoride, such as the dose, duration, and timing during the period of enamel formation [1]. Some studies have also shown that there is a greater risk of dental fluorosis incidence if there is an excessive amount of fluoride in the environment during the first two years of life, which are considered a critical period, where susceptibility is higher [64]. That is why the risk of dental fluorosis incidence after a professional local application of fluoride compounds is quite low.

As a future objective, it is necessary to add alternatives to fluoride, in order to have a natural supply of hydroxyapatite not aggravating the environmental factors. Toothpastes containing biomimetic hydroxyapatite Zn-carbonate hydroxyapatite (microRepair^®^, Coswell SPA, Bolonia, Italy) have been investigated in recent years and displayed a significant re-mineralizing activity [52]. Non-calcium-based technologies focus primarily on pH modification within the saliva, and surface biofilm. An example includes xylitol. Likewise, the activity of the trimetaphosphate ion (TMP) to enhance enamel and dentin remineralization is also currently an area of active research interest [65]. The following are calcium-based re-mineralizing agents: amorphous calcium phosphate (ACP) [66]; casein phosphopeptide (Recaldent) [67]; functionalized tricalcium phosphate (fTCP); particulate bioglass (NovaMin) [68]; nano-hydroxyapatite [69].

## 5. Conclusions

The findings of this study revealed an increase in the occurrence and severity of fluorosis in people living in areas in which drinking water has a low fluoride ion content. Associating fluorosis exclusively with elevated levels of fluoride ions in drinking water is not justified nowadays, as there are many other potential sources of fluoride.

The availability of fluoride from other sources has increased significantly, and fluoride in drinking water is now recognized as just one component making up an individual’s total fluoride intake. Because of the widespread use of toothpaste and other dental health care products containing fluoride and the potential for fluoride exposure from several other sources, monitoring the total exposure to which we are subjected has become essential. As a future objective, it is necessary to add the alternatives to fluoride, to have a natural supply of hydroxyapatite and as to not aggravate the environmental factors. Many opportunities and challenges lie ahead in the implementation of new remineralization technology.

It is beneficial to remember the possibility to use toothpastes that do not contain fluoride. Instead, it is replaced with natural components such as herbs, which are characterized by anti-bacterial properties, as seen in e.g., bamboo salt [70]. Other types of toothpaste include those which contain phosphorus compounds, calcium phosphate, functionalized β-tricalcium phosphate, calcium glycerophosphate, cyclophosphates, calcium sucrose phosphate, zinc hydroxyapatite, or toothpastes with an acidic pH [71,72]. Recently, it has been shown that toothpaste with a potent penetration promoter, azone, and vitamin D3 may be helpful in the prevention of the common deficiencies of this vitamin. According to the authors, the aforementioned toothpaste did not contain fluoride compounds [73].

## Figures and Tables

**Table 1:** The severity of fluorosis in the study group, according to Dean’s Index.

Severity of Fluorosis *	Number of Cases in Groups with Different Severity Level * *n* (%)	
0	607 (87.2%)	
1	12 (1.7%)	Total groups 1–3: 89 (12.8%)
2	56 (8.1%)	
3	21 (3.0%)	
4	n.o.	
5	n.o.	
Total number of study participants:	696	

* According to the Dean’s index. n.o.—not observed.

**Table 2:** A quantitative analysis of total protein in teeth enamel in groups with different stages of fluorosis.

Group	*n*	Mean ± SD (mg/g)	*p*-Value
I	10	28.12 ± 1.75	* *p* = 0.002
II	10	32.08 ± 2.12	** *p* = 0.914
III	10	33.01 ± 3.12	*** *p* < 0.001

*p*-values obtained with the post hoc Tukey HSD test: * I vs. II, ** II vs. III, *** I vs. III. Statistically significant values were marked in bold. SD—standard deviation.

**Table 3:** Fluoride content in tooth enamel in groups with different stages of fluorosis.

Group	*n*	Mean ± SD (mg/g)	*p*-Value
I	10	0.81 ± 0.10	* *p* < 0.001
II	10	1.17 ± 0.13	** *p* = 0.221
III	10	1.24 ± 0.22	*** *p* < 0.001

*p*-values obtained with the post hoc Tukey HSD test: * group I vs. II, ** II vs. III, *** III vs. I. Statistically significant values were marked in bold. SD—standard deviation.

## Data Availability

The datasets generated during the current study are available from the corresponding author on reasonable request.
